# QTL Mapping of Zeaxanthin Content in Sweet Corn Using Recombinant Inbred Line Population across Different Environments

**DOI:** 10.3390/plants12193506

**Published:** 2023-10-09

**Authors:** Yahui Zhang, Yunqi Tang, Weicai Jin, Yu Liu, Guangyu Li, Wenhao Zhong, Jun Huang, Wenyi Wang

**Affiliations:** College of Agriculture, South China Agricultural University, Guangzhou 510642, China; yahuiscau@163.com (Y.Z.); yqtang@stu.scau.edu.cn (Y.T.); xdfjinweicai@163.com (W.J.); yuliu2019@126.com (Y.L.); liguangyu2105@163.com (G.L.); whwh@stu.scau.edu.cn (W.Z.); junhuang@scau.edu.cn (J.H.)

**Keywords:** RIL population, zeaxanthin, QTL mapping, sweet corn, LOD

## Abstract

Zeaxanthin is a naturally occurring xanthophyll carotenoid obtained from diet sources. Particularly, sweet corn is a major source of dietary zeaxanthin. To investigate the genetic basis of zeaxanthin content regulation in sweet corn, a recombinant inbred line (RIL) population comprising 191 families was constructed using two inbred lines (K44 and F22) with contrasting zeaxanthin content in the grain. The zeaxanthin content in the dry grains of this population grown at different locations was determined using high performance liquid chromatography (HPLC). Subsequently, 175 polymorphic simple sequence repeat (SSR) markers were used to construct a linkage map with a total length of 4322.37 cM and with an average distance of 24.4 cM. A total of eight QTLs located on chromosomes 4, 5, 7, 9, and 10 were detected. The QTLs located in umc1632-umc1401 on chromosome 7 were detected in different environments and explained 11.28–20.25% of the phenotypic variation, implying it is the main QTL controlling zeaxanthin content in the dry grains of sweet corn. Collectively, the present study provides a genetic map and theoretical guidance for the cultivation of sweet corn varieties with a high zeaxanthin content.

## 1. Introduction

Zeaxanthin is one of the most common naturally occurring carotenoids in plants including yellow corn, spinach, squash, and oranges [1]. High dietary ingestion of zeaxanthin and lutein and elevated serum levels of these xanthophylls are associated with a lower risk of age-related macular degeneration (AMD) [2], which is the most common cause of blindness [3,4]. Zeaxanthin and lutein are absorbed and accumulate in the human eye (macula and retina), where they function as light filters, protecting eye tissues from sunlight damage through their antioxidant activity [5]. Dietary supplementation with zeaxanthin and lutein reduced the progression of AMD by approximately 10% [6]. Zeaxanthin and lutein are now the standard of care for decreasing the probability of advanced AMD [7,8,9,10]. Other health benefits of zeaxanthin include reduced risk of atherosclerosis [11], anti-inflammatory effects [12], breast cancer [13], head and neck cancer [14]. As a carotenoid xanthophyll pigment, zeaxanthin is responsible for the characteristic yellow and orange in plants and food [15,16].

**Figure 1:**
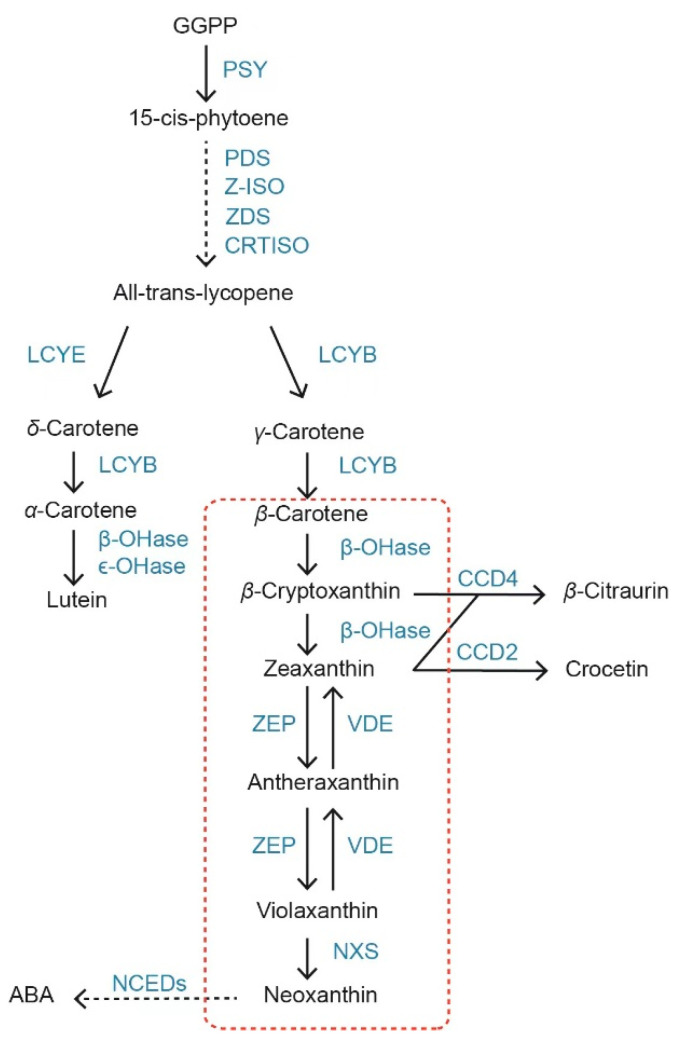
Zeaxanthin metabolic pathway in plants. Enzymatic reactions are represented by arrows. The dashed arrow represents several enzymatic reactions. Enzymes are indicated in blue text. GGPP, geranylgeranyl diphosphate; PSY, phytoene synthase; PDS, phytoene desaturase; ZDS, ζ-carotene desaturase; CRTISO, carotenoid isomerase; ζ-carotene isomerase; LCYE, lycopene ε-cyclase; LCYB, lycopene β-cyclase; β-OHase, β-carotene hydroxylase; ε-OHase, ε-carotene hydroxylase; ZEP, zeaxanthin epoxidase; VDE, violaxanthin de-epoxidase; NXS, neoxanthin synthase; CCD, carotenoid cleavage dioxygenase; NCED, 9-cis-epoxycarotenoid dioxygenase; and ABA, abscisic acid.

Owing to the importance of carotenoids, the regulation of zeaxanthin synthesis and its accumulation in plants has been extensively studied. The carotenoid biosynthetic pathway, which starts with the formation of phytoene from geranylgeranyl pyrophosphate (GGPP) mediated by phytoene synthase (PSY) [17,18]. PSY has been shown to determine the flux of the pathway, thus affecting the level of total carotenoids. All trans lycopene was obtained by 15-cis-phytoene under the action of phytoene desaturase (PDS), ζ-carotene isomerase (Z-ISO), ζ-carotene desaturase (ZDS), and carotene isomerase (CRTISO). Lycopene β-cyclase (LCYB) and lycopene ε-cyclase (LCYE) catalyze the cyclization reactions to produce orange-colored γ-carotene and β-carotene, respectively (Figure 1). β-carotene can be subsequently hydroxylated by β-carotene hydroxylase (CRTRB) in a two-step reaction with zeaxanthin, with β-cryptoxanthin as an intermediate product. Over the past few decades, the metabolic engineering of carotenoid biosynthesis has been employed to increase carotenoid content in plants. 

Several attempts to control the expression of β-carotene hydroxylase and lycopene β-cyclase genes to assist xanthophyll collection in tomato fruit have been previously reported [19,20,21,22,23]. For example, concurrent overexpression of *lycopene β-cyclase (LCYB)* and *carotene beta-hydroxylase 1 (BCH1, CrtR-b1)* did not lead to any appreciable rise in the xanthophyll content of ripe fruits compared to the overexpression of only the *LCYB* transgene [23]. Overexpression of *carotene beta-hydroxylase 2 (BCH2, CrtR-b2)* in tomato fruits produces free violaxanthin and a significant amount of esterified xanthophylls in hemizygous transgenic plants during the immature green stage [19]. A bacterial β-carotene hydroxylase was expressed in tobacco, and the results showed that zeaxanthin was formed more rapidly and to a greater extent when low-light-adapted transgenic plants were transferred to high light conditions than in wild-type plants [24]. Recently, Lee et al. silenced the zeaxanthin epoxidase (ZEP) gene, resulting in a significant change in the ratios of zeaxanthin in peppers [25].

Zeaxanthin is not synthesized in the human body and must therefore be obtained from dietary sources. The accessibility of zeaxanthin in food is relatively poor and green leafy vegetables contain negligible or no zeaxanthin [26]. Some fruits, such as oranges, peppers, mango, and papaya contain zeaxanthin within the range of 2–10 μg/g fresh weight (FW) [16,26,27]. In certain varieties of orange peppers, paprika, and Goji berries (Lycium fruit), zeaxanthin can reach 600–800 μg/g dry weight (DW) [28,29,30,31,32,33]. Compared to most plant-derived dietary sources, yellow sweet corn is an important source, and its product is an important contributor to dietary intake. A series of quantitative trait loci (QTLs) that control zeaxanthin content have been reported in maize over the past few decades. For example, three zeaxanthin-related QTLs were detected on chromosomes 6, 7, and 8, explaining 6.5–19.8% of the phenotypic variation in maize kernels [34]. Similarly, to identify major loci controlling carotenoid content in maize grains, a genetic linkage map was constructed from 233 recombinant inbred lines by crossing maize inbred lines By804 and B73, and a total of 31 putative QTLs, including 23 for individual and 8 for total carotenoids, were detected; furthermore, five QTLs associated with zeaxanthin were located on chromosomes 1, 6, 8, and 10, which accounted for 3.8–16.6% of the phenotypic variation [35]. Subsequently, the major-effect QTL *qZea6a* explained 41.4–71.4% of the phenotypic variation, and two more QTLs, *qZea4a* and *qZea3a* showed LOD  >  3 for zeaxanthin concentration in two generations and three different environments [36]. In short, few studies have detected zeaxanthin-related QTLs in maize and, although it is a particularly good source of zeaxanthin, knowledge of zeaxanthin regulation in sweet corn remains limited. 

The objectives of this study are to identify the QTLs responsible for controlling zeaxanthin content in sweet corn using a RIL population comprising 191 families constructed with two inbred lines. Our findings provide information on the potential to facilitate breeding for improved zeaxanthin levels in sweet corn grain.

## 2. Results

### 2.1. Construction of the Zeaxanthin Calibration Curve

**Figure 2:**
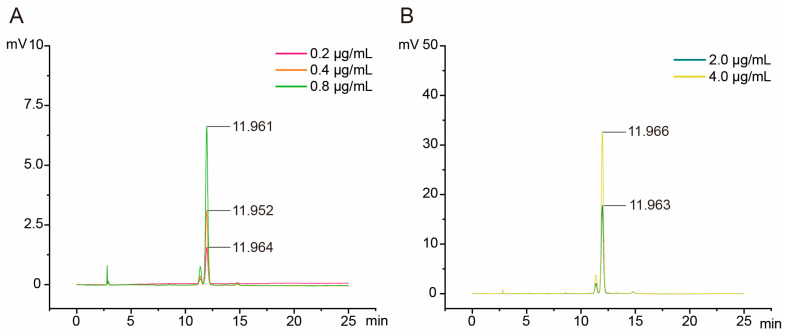
Chromatograms obtained for zeaxanthin standard solutions. (**A**) The 0.2, 0.4, and 0.8 μg/mL zeaxanthin standard solutions; (**B**) 2.0 and 4.0 μg/mL zeaxanthin standard solutions.

Using LC16 high performance liquid chromatography (HPLC), the 0.2, 0.4, 0.8, 2.0, and 4.0 μg/mL zeaxanthin standard solutions were qualitatively and quantitatively determined at a wavelength of 450 nm. An obvious single peak was detected at approximately 11.96 min after sample injection, and the height and area of the peak changed with concentration (Figure 2). Therefore, zeaxanthin retention time was approximately 11.96 min (Figure 2). The linear relation (y = 0.000008x − 0.0081) between the zeaxanthin concentration (y) and the area of the absorption peaks (x) was obtained based on the concentration of standard solutions and the corresponding area of absorption peaks (R^2^ = 0.9978) (Table 1).

### 2.2. Analysis of Zeaxanthin Content in the RIL Population

**Figure 3:**
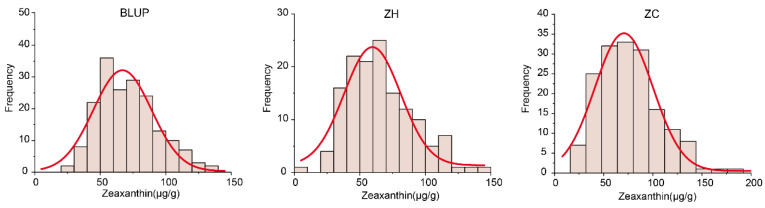
Frequency distribution of zeaxanthin content. BLUP, best linear unbiased prediction; ZH, Zhuhai; and ZC, Zengcheng.

The zeaxanthin content in dry corn grains of the RIL population grown at different locations, including Zengcheng and Zhuhai, was detected using HPLC. In the Zengcheng environment, the lowest and highest zeaxanthin contents were 18.66 and 186.14 μg/g, respectively, the difference being almost ten-fold. The mean value was 76.32 μg/g (Table 2). The skewness and kurtosis were also examined (Table 2). In turn, in the Zhuhai environment, the lowest zeaxanthin content was 9.57 μg/g, and the highest was 146.93 μg/g. In this case, the mean content was 65.98 ± 25.51 μg/g (Table 2, Figure 3). These results showed that zeaxanthin content varied widely in the population and that the QTL loci regulating zeaxanthin content was effectively identified and mined. Analysis of variance revealed that differences caused by genotype and environment were highly significant and suitable for QTL mapping (Table 3). 

### 2.3. Construction of the Genetic Map of the RIL Population

A total of 1052 pairs of SSR markers were used to screen for polymorphisms between parent inbred lines K44 and F22. In all, 233 markers showed polymorphisms between the parents. Of these, 175 polymorphic markers with clear bands and good amplification effects accounted for 78.9% and 16.7% of the polymorphic markers and the total number of markers, respectively. Notably, 42 polymorphic markers exhibited segregation distortions. 

**Figure 4:**
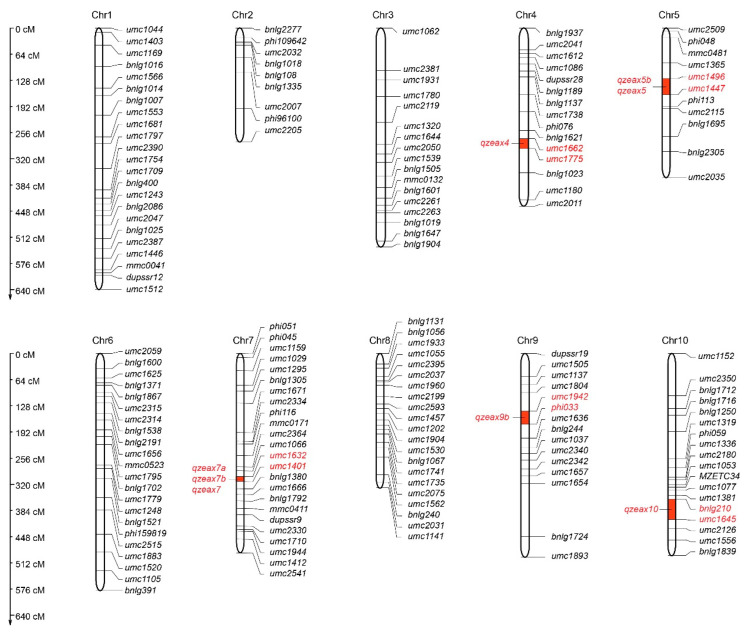
Genetic linkage map and locations of putative QTL for zeaxanthin detected in 191 RILs derived from a cross between inbred lines K44 and F22 differing for zeaxanthin content. The genetic distance between markers is given in cM, and the symbols in the linkage map indicate the position of QTLs.

A genetic map of the RIL population was constructed using 175 SSR polymorphic markers. The total length of the map was 4322.37 cM, and the average distance between the SSR markers was 24.4 cM. The length range of the linkage group was 280.42–642.90 cM, the shortest was chromosome 2, the longest was chromosome 1, and the average distance between markers was 15.76–33.43 cM (Table 4, Figure 4). There were five intervals larger than 80 cM, such as umc2007-phi96100 and phi96100-umc2205 on chromosome 2, umc1062-umc2381 on chromosome 3, umc1654-bnlg1724 on chromosome 9, and umc1152-umc2350 on chromosome 10 (Table 4, Figure 4). Based on the comparison of the sequences and loci of the molecular markers, the distribution of markers on the chromosomes was relatively uniform, and the linkage map was highly consistent with the integrated map of maize published in the genome database, which may be used for further QTL mapping.

### 2.4. QTL Mapping of Zeaxanthin in the RIL Population

**Figure 5:**
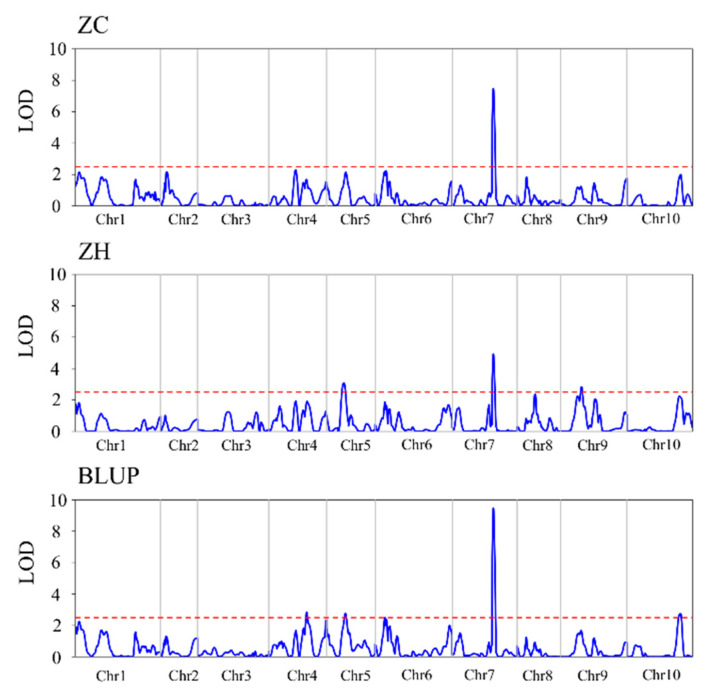
QTL mapping of zeaxanthin. ZC, Zengceng; ZH, Zhuhai; BLUP, best linear unbiased prediction; and LOD, limit of detection.

A zeaxanthin-related QTL was detected in dry corn grains in the Zengcheng environment (Table 5, Figure 5). The *qZeax7a* is located between markers umc1632 and umc1401 on chromosome 7, with an additive effect of −14.46 and an LOD value of 7.48, thus explaining 20.25% of the phenotypic variation for zeaxanthin.

Meanwhile, in the Zhuhai environment, three zeaxanthin-content-related QTLs were detected (Table 5, Figure 5), located on chromosomes 5, 7, and 9. *qZeax5b* is located between markers umc1496 and umc1447 on chromosome 5, with an additive effect of 9.27 and an LOD value of 3.08, explaining 8.8% of the zeaxanthin phenotypic variation. *qZeax7b* is located between markers umc1632 and umc1401 on chromosome 7, with an additive effect of 10.48 and an LOD value of 4.92, explaining 11.28% of the zeaxanthin phenotypic variation. *qZeax9b* is located between the markers umc1942 and phi033 on chromosome 9, with an additive effect of −9.93 and an LOD value of 2.84, explaining 10.13% of the zeaxanthin phenotypic variation.

The best phenotypic linear unbiased prediction (BLUP) was used to detect zeaxanthin content-related QTLs. Four QTLs were identified, which were located on chromosomes 4, 5, 7, and 10 (Table 5, Figure 5). *qZeax4* is located between markers umc1662 and umc1775 on chromosome 7, with an additive effect of 7.10 and an LOD value of 2.83, thus explaining 5.91% of the phenotypic variation for zeaxanthin content. *qZeax5* is located between markers umc1496 and umc1447 on chromosome 5, with an additive effect of 8.82 and an LOD value of 2.76, explaining 9.21% of the zeaxanthin phenotypic variation. *qZeax7* is located between markers umc1632 and umc1401 on chromosome 7, with an additive effect of −11.15 and an LOD value of 9.47, explaining 14.74% of the zeaxanthin phenotypic variation. *qZeax10* is located between markers bnlg210 and umc1645 on chromosome 10, with an additive effect of 6.08 and an LOD value of 2.74, explaining 4.43% of the zeaxanthin phenotypic variation.

Therefore, eight QTLs related to zeaxanthin content were detected in Zengcheng, Zhuhai, and BLUP, which are located on chromosomes 4, 5, 7, 9, and 10. QTLs located in the interval of chromosomes umc1496-umc1447 were detected in both Zengcheng and Zhuhai environments (Table 5, Figure 5). Notably, QTLs located in umc1632-umc1401 on chromosome 7 were detected in Zengcheng, Zhuhai, and BLUP (Table 5, Figure 5). In turn, QTLs located in the umc1942-phi033, umc1662-umc1775, and bnlg210-umc1645 intervals were detected only in the Zhuhai or BLUP values (Table 5, Figure 5). Moreover, the QTL located in umc1632–umc1401 on chromosome 7 explained 11.28–20.25% of the phenotypic variation and were detected in Zhuhai, Zengcheng, and BLUP values (Figure 5), indicating that the QTL in this interval is the main QTL controlling zeaxanthin content in dry sweet corn grains.

## 3. Discussion

Zeaxanthin, the most common carotenoid, exists primarily in the human eye, liver, spleen, kidneys, and other organs. Neither humans nor animals are able to synthesize zeaxanthin independently but can only obtain the required zeaxanthin through diet. Corn is one of the few sources of zeaxanthin along with its isomer, lutein, and together, they constitute the major carotenoids contributing to the characteristic color of sweet corn. Previous studies have shown that the zeaxanthin content of yellow corn is higher than that of white corn [37]. The metabolic engineering of zeaxanthin has been achieved in several plants. In potato (*Solanum tuberosum* L.), the concentration of zeaxanthin in tubers was significantly increased by silencing zeaxanthin epoxidase (ZEP) [38]. Transgenic expression of *Psy1* in a white corn variety, crtI from the bacteria *Pantoea ananatis,* and LCYB and BCH from *Gentiana lutea* increased zeaxanthin levels in seeds dramatically [39]. In tomato, the increased accumulation of zeaxanthin in fruit was achieved by the transgenic expression of LCYB and BCH [40]. Karniel et al. (2020) demonstrated that carotenoid biosynthesis in tomato fruit can be successfully manipulated nontransgenically to achieve zeaxanthin accumulation at a concentration of 39 μg/g FW (equal to 650 μg/g dry weight) [41].

Population size and density of molecular markers had an impact on QTL mapping. If the molecular marker density is too small and the marker interval is large, it will lead to inaccurate QTL mapping results. Conversely, if the molecular marker density is too large and the marker interval is small, it may cause interference between adjacent QTL, resulting in deviation of QTL mapping results. Therefore, QTL mapping resolution is largely determined by the size of confidence interval of QTL. In the present study, a recombinant inbred line (RIL) population comprising 191 families was constructed using two inbred lines (K44 and F22) for contrasting zeaxanthin content in the grain. The RIL population originates from several generations of recombination, providing greater opportunities for linkage breakdown and separation of linked genes and markers [42]. Previous studies have shown that, if the distance between two molecular markers does not exceed 50 cM, the effect and position of the detected QTL are acceptable [43]. Chander et al. (2008) identified 31 putative QTL including 23 for individual and 8 for total carotenoids in maize grain using 233 recombinant inbred (RI) populations with 201 molecular markers [35]. Jittham et al. (2017) used single nucleotide polymorphism (SNP) markers to detect carotenoid-related QTL in 178 RIL populations [44]. Here, a maize linkage genetic map with a total length of 4322.37 cM was constructed with 175 pairs of primers, covering 10 linkage groups of maize. The resulting average distance between SSR markers was 24.4 cM, which can be used for gene mapping. 

In this study, 42 polymorphic markers showed segregation distortion. The phenomenon of partial segregation in potato and other species has been previously reported [45]. For example, Khedikar et al. (2010) constructed a recombinant inbred line comprising 268 families using TAG24 × GPBD4. A genetic map was constructed using 67 polymorphic markers, of which 20 pairs of SSR primers deviated, and partial segregation markers accounted for 29.85% of the total markers [46]. Similarly, in a population of 146 recombinant inbred lines constructed using TG26 × GPBD4, 53 polymorphic markers were used to construct genetic maps, of which 15 pairs deviated, accounting for 28.3% of the total number of markers [47]. Chi-square tests were performed on 175 pairs of SSR markers used for population genotype identification. We found that 33 pairs of SSR molecular markers had significantly biased segregation, and 9 pairs of SSR markers had highly and significantly biased segregation. Partial segregation occurred in 42 pairs of SSR molecular markers, accounting for 24% of the total SSR markers, which was not particularly significant and did not affect the experimental results.

Zeaxanthin and lutein are the major carotenoids in corn. However, only a few QTLs related to zeaxanthin have been identified to date [48]. The PS1 locus on chromosome 5 encodes LCYB. This gene is thought to be essential for zeaxanthin accumulation in maize [49]. The ZEP1 gene, which controls the zeaxanthin epoxidase gene, is one of the major genes involved in the carotenoid metabolic pathway in maize [50,51]. The QTL detected on maize chromosomes 6, 8, and 10 explained 12.5%, 6.7%, and 19.4% of the phenotypic variation in zeaxanthin, respectively [44]. It was reported that the quantitative trait loci (QTL) for zeaxanthin on chromosome 6 account for 13.9–16.3% of the phenotypic variation [34,35]. Dong et al. found that the QTL *qZea6a* on chromosome 6 accounts for 41.4–71.4% of the phenotypic variation [36]. This might be due to the large genetic differences in zeaxanthin genes between the two parents. In addition, alleles producing higher zeaxanthin concentrations were the progeny of parents with high zeaxanthin-production abilities. Therefore, parents with high zeaxanthin concentrations are extremely important for improving zeaxanthin production in maize breeding programs.

The *ZEP1* gene, which controls the zeaxanthin epoxidase gene, is one of the major genes involved in the carotenoid metabolic pathway in maize [50,51]. The QTL detected on maize chromosomes 6, 8, and 10 explained 12.5%, 6.7%, and 19.4% of the phenotypic variation in zeaxanthin, respectively [44]. In the present study, zeaxanthin-content-related QTLs were identified across different environments. Analysis of variance revealed that zeaxanthin content differences caused by genotype and environment were highly significant. *qZeax4* and *qZeax10* were detected only in BLUP and *qZeax9b* under Zhuhai conditions (Figure 4, Table 5), suggesting that the environment affected zeaxanthin-content-related QTL detection. Previous studies have shown that the maize genome regions bin 3.04, 4.03~4.04, 6.01~6.02, 7.00~7.02, 8.03~8.05, and 10.05~10.06 were correlated with zeaxanthin concentration [34,35]. The *qZeax7* located in umc1632–umc1401 on chromosome 7 explained 11.28–20.25% of the phenotypic variation and was detected in Zhuhai, Zengcheng, and BLUP (Figure 5, Table 5), suggesting that *qZeax7* has major effects on zeaxanthin in sweet corn. Notably, the interval between *qZeax7* and *qZeax10* was consistent with the previous detection interval [34,35]; however, no consistent interval was found between *qZeax4* and *qZeax5*, indicating that *qZeax4* and *qZeax5* are new QTLs related to zeaxanthin content control. 

To investigate the locus-controlling zeaxanthin content in sweet corn, a linkage map based on RIL populations was constructed, and several QTLs were identified across different environments. The major-effect QTL *qZeax7* potentially provides new genetic resources for the development of new sweet corn varieties with a high zeaxanthin content. Nonetheless, further research is necessary to clone the candidate genes, which may facilitate plant genetic engineering for enhanced zeaxanthin content.

## 4. Materials and Methods

### 4.1. Plant Material, Population Construction, and DNA Extraction

A high zeaxanthin concentration maize inbred line K44 and a low zeaxanthin concentration maize inbred line F22 were selected as parents to generate the K44 × F22 cross. All inbred lines were provided by the Sweet Corn Research Group of the South China Agricultural University (SCAU). In September 2018, K44 and F22 were planted at the Zengcheng Experimental Base (23.42° N, 113.35° E) of the SCAU. Using K44 as the male parent and F22 as the female parent, F_1_ seeds of the hybrid combination were obtained. In March 2019, F_1_ seeds were planted at the Zengcheng Experimental Base. F_2_ seeds were obtained via F_1_ single-seed sowing and self-crossing. Subsequently, F_2_ seeds were planted at the Zengcheng Experimental Base, and single-seed sowing, self-pollination, and single-plant harvest were performed, and F_3_ seeds were obtained. In March 2020, the harvested F_3_ seeds were planted at the Zengcheng Experimental Base for self-pollination. In September 2020, the harvested F_4_ seeds were planted for self-pollination. In September 2021, the harvested F_6_ seeds were planted at the Zengcheng Experimental Base and Zhuhai (22.15° N, 113.37° E) Experimental Base of SCAU. Leaves were collected from the parents and 191 families, and DNA was extracted using the CTAB method.

### 4.2. Extraction and Determination of Zeaxanthin

Mature dried corn grains were ground into a powder, and 1 g samples were weighed and transferred to 50 mL tubes. HPLC grade methanol was added to 20 mL and ultrasonicated for 30 min at 40 kHz with 80% power. The mixture was immediately centrifuged at 10,000 rpm for 5 min. The supernatant was retrieved and transferred to 50 mL amber volumetric flasks. Samples were then filtered (0.22 µm syringe filter) and placed into HPLC vials, prior to HPLC analysis.

The samples were analyzed using a Shimadzu LC16 HPLC; briefly, 20 μL of extract was injected onto a YMC C30 carotenoid Column—5 μm, 4.6 mm × 250 mm. The mobile phase consisted of acetonitrile (75%)–methanol (25%) at a flow rate of 1 mL/min and a column temperature of 35 °C. The detection wavelength was 445 nm, and the single detection time was 25 min.

### 4.3. Construction of the Zeaxanthin Standard Curve

Zeaxanthin (10 mg) was dissolved in methanol to obtain a final volume of 100 mL. Then, 0.02, 0.04, 0.08, 0.20, and 0.40 mL of zeaxanthin standard solutions were placed in 10 mL volumetric flasks, diluted with methanol to volume, and mixed. Standard solutions with concentrations of 0.2, 0.4, 0.8, 2.0, and 4.0 μg/mL were obtained. The standard samples were then filtered (0.22 µm syringe filter) and placed into HPLC vials.

### 4.4. Genotyping and Analysis

In all, 1052 pairs of detected SSR markers were screened for polymorphisms in the parental inbred lines K44 and F22. The results showed 175 pairs of polymorphic markers with significant differences and clear bands were obtained. These 175 pairs of polymorphic markers were used to identify population genotypes. The PCR reaction system is 15 μL, including 2 μL of template DNA, 1.2 μL of forward primer and 1.2 μL of reverse primer, 5.3 μL of PCR mix, and 5.3 μL of ddH_2_O. The PCR conditions consisted of 95 °C for 5 min, 35 cycles of 95 °C for 30 s, 55 °C for 30 s, and 72 °C for 30 s, as well as a final extension of 5 min at 72 °C. After PCR, the amplified fragments were separated by polyacrylamide gel electrophoresis (PAGE). The QTL IciMapping software (https://isbreedingen.caas.cn/, accessed on 1 December 2021) was used to analyze the molecular markers of the population.

### 4.5. Data Processing and Statistical Analysis

According to the requirements of the QTL IciMapping software, the banding pattern consistent with parental line K44 was 0, the banding pattern consistent with parental line F22 was 2, and the heterozygosity and deletion values were −1. The Kosambi mapping function was selected using the Map program to construct a genetic map. The link between SSR molecular markers and phenotypes in the RIL population was analyzed using the bip function, and the limit of detection (LOD) value was set to 2.5. Phenotypic data were recorded in Excel, and the R-packet was used for best linear unbiased prediction (BLUP), analysis of variance, and related pictures.

## Figures and Tables

**Table 1:** Standard solution and absorption peak area of zeaxanthin.

Concentration (μg/mL)	Time (min)	Area (mv * s)
0.2	11.964	22,149
0.4	11.952	45,202
0.8	11.961	96,548
2.0	11.963	257,506
4.0	11.966	475,438

**Table 2:** Statistical analysis of zeaxanthin content.

Environment	Population	Min	Max	Mean	SD	Skewness	Kurtosis
Zengcheng	166	18.66	186.14	76.32	30.43	0.69	0.52
Zhuhai	141	9.57	146.93	65.98	25.51	0.65	0.19
BLUP	182	27.49	139.02	70.88	22.53	0.56	−0.07

Min: minimum value; Max: maximum value; and SD: standard deviation.

**Table 3:** Analysis of variance of zeaxanthin content in different environments.

Variation Source	DF	SS	MS	F	*p*-Value
Genotype	183	452,722.25	2473.89	1455.25	<0.0001
Environment	1	16,713.75	16,713.75	9831.73	<0.0001
Genotype × Environment	126	54,120.81	429.53	252.67	<0.0001
Error	309	525.29	1.69		
Total	619	524,082.31			

DF: degree of freedom; SS: sum of squares; and MS: mean square.

**Table 4:** Distribution of SSR markers on 10 chromosomal linkage groups.

Chromosome	Genetic Distance (cM)	Marker Number (Pairs)	Mean Distance (cM)
Chr1	642.90	23	27.95
Chr2	280.42	9	31.16
Chr3	538.21	17	31.66
Chr4	437.68	15	29.18
Chr5	367.73	11	33.43
Chr6	582.61	22	26.48
Chr7	489.34	24	20.39
Chr8	330.96	21	15.76
Chr9	500.41	15	33.36
Chr10	496.97	18	27.61

**Table 5:** QTL mapping of zeaxanthin content in Zengcheng and Zhuhai Environments and BLUP.

Environment	Chromosome	QTL	Marker Interval	LOD	PVE (%)	ADD
ZC	7	*qZeax7a*	umc1632-umc1401	7.48	20.25	−14.46
ZH	5	*qZeax5b*	umc1496-umc1447	3.08	8.80	9.27
	7	*qZeax7b*	umc1632-umc1401	4.92	11.28	−10.48
	9	*qZeax9b*	umc1942-phi033	2.84	10.13	−9.93
BLUP	4	*qZeax4*	umc1662-umc1775	2.83	5.91	7.10
	5	*qZeax5*	umc1496-umc1447	2.76	9.21	8.82
	7	*qZeax7*	umc1632-umc1401	9.47	14.74	−11.15
	10	*qZeax10*	bnlg210-umc1645	2.74	4.43	6.08

LOD: limit of detection; PVE: the proportions of phenotypic variance; ADD: additive effects; and QTL: quantitative trait locus.

## Data Availability

The data is contained within the manuscript.
